# Prevalence of anxiety symptoms, depression and temporomandibular dysfunction in prisoners and workers from a brazilian prison: an observational study

**DOI:** 10.4314/ahs.v25i1.47

**Published:** 2025-03

**Authors:** José Carlos de Oliveira Gomes Filho, Lucian Lacerda Franco Rocha Rodrigues, Thiago Bezerra Leite, Flávia Dayana Ribeiro da Silveira, Celiane Mary Carneiro Tapety, António Sérgio Guimarães

**Affiliations:** 1 São Leopoldo Mandic College of Dentistry, Orofacial Pain Laboratory, Campinas, SP, Brazil; 2 Federal University Ceará, Pharmacy, Dentist and Nursing School, Fortaleza, CE, Brazil; 3 Leão Sampaio University Center, Juazeiro do Norte, Brazil; 4 Unifacid Wyden, Teresina, Brazil; 5 Federal University Ceará, School of Dentistry, Sobral, CE, Brazil

**Keywords:** Temporomandibular dysfunction, Depression, Anxiety, Prisoners

## Abstract

**Background:**

Temporomandibular Disorder (TMD) is a condition in which there are several psychosomatic and/or psychosocial aspects. Imprisonment situations may be an example of a triggering factor for stress and TMD symptoms, especially if taken into account that the incarcerated population is bigger than the general population.

**Objective:**

The present study aims to investigate the prevalence of symptoms of anxiety, depression and temporomandibular disorder (TMD) in prisoners and employees of a closed male penitentiary in Brazil.

**Methods:**

A total of 140 prisoners and 50 employees were part of the study. This is a cross-sectional study with a quantitative approach, where the symptom questionnaire for Temporomandibular Disorders (DC/TMD) and the Hospital Anxiety and Depression Scale (HAD) were applied.

**Results:**

The anxiety and depression levels found in prisoners were 64.86% and 48.64%, respectively. There was a higher statistical significance in the reported symptoms of headache in employees with anxiety when compared to those without anxiety (p= 0.03). Regarding depression, it was observed that it influenced the symptoms of jaw opening and locking, both in employees (p=0.09) and inmates (p=0.01).

**Conclusion:**

A high prevalence of TMD symptoms, anxiety, and depression were identified in both prisoners and employees, especially the prisoners.

## Introduction

Temporomandibular disorder (TMD) is a complex condition within a biopsychosocial disease model, and which, in most cases, is not a pathology only located in the orofacial region (Ohrbach, Dworkin, 2016). TMD symptoms include decreased range of mandibular movement, functional limitation of the mandible, pain in the orofacial muscles, and in the temporomandibular joint (TMJ), noises or deviation during mouth opening (Liu, Steinkeler, 2013). TMD is one of the most common chronic musculoskeletal diseases. Due to its relation to pain, it can disturb the daily activities of the affected individual, as well as their psychosocial functioning and their quality of life. For this very reason, it is important to accurately diagnose this condition so that clinical care is carried out (Ahmad, Schiffman, 2016; Bitiniene et al., 2018). This condition may be caused by muscle hyperfunction or parafunction, traumatic injuries, hormonal influences, and joint changes (Liu, Steinkeler, 2013). Some studies also associate TMD with anxiety, stress and depression (Nazeri et al., 2018; Sójka et al., 2019). Some studies also associate TMD with anxiety, stress and depression (Nazeri et al., 2018; Sójka et al., 2019). In order to identify symptoms and diagnosis, the DC/TMD protocol (Diagnostic Criteria for Temporomandibular Disorders) is the only evidence-based TMD diagnostic system that has undergone rigorous scientific investigation. Its use, both in clinical settings and in scientific research, allows the standardization of criteria for decision-making diagnoses and the identification of patients with a variety of TMD presentations, from simple to more complex cases (Schiffman et al., 2014; Ohrbach, Dworkin, 2016). This protocol was validated for the Portuguese language in 2016 (Ohrbach, 2016).

Incarcerated individuals often experience psychological distress, such as anxiety and depression, and there is a lack of interventions to deal with this situation (López-Pérez et al., 2018). The prevalence of self-reported TMD symptoms and diagnosed TMD symptoms has been considered high in prisoners (Vainionpää et al., 2018).

In this context, considering that prisons are stressful environments, for both prisoners and workers, given the scarcity of work with the incarcerated population, this study aims to assess the prevalence of TMD symptoms, as well as depression and anxiety among inmates and employees of a closed male penitentiary located in Brazil.

## Materials and methods

### Type of study

This work is a cross-sectional, descriptive-exploratory study, with a quantitative approach that followed the STROBE checklist.

### Ethical aspects

The study was carried out following the ethical resolutions, respecting the principles of beneficence, non-maleficence, autonomy and justice. It was approved by the Ethics and Research Committee of Sào Leopoldo Mandic College (Campinas, Brazil) under protocol number: 5.257.823.

### Research location and Study population

For the study, individuals under deprivation of liberty in a Brazilian penitentiary (Bom Jesus, PI, Brazil) were evaluated. The sample calculation for a finite population was used, stratified by proportion. The population consisted of 140 prisoners and 50 employees, with a sampling error of 5% and a confidence level of 95%. Prisoners with more than six months of imprisonment who voluntarily agreed to participate in the study and answer the questionnaires were included. Prisoners with general inflammatory diseases of the connective tissue (e.g. rheumatoid arthritis) and prisoners with symptoms that could be attributed to other diseases of the orofacial region (e.g. toothache, neuralgia) were not included in the sample.

Those who refused to answer the questions or sign the Informed Consent Form (ICF) were excluded. In order to make comparisons, data was collected with people who work in the same prison too.

### Data collection

The application of the DC/TMD and of the Hospital Anxiety and Depression (HAD) questionnaires was done by signing the ICF at the place and time authorized by the penitentiary board, respecting the local prison safety rules and COVID-19 prevention rules, such as the use of PFF2 masks without valve and sanitize hands with alcohol gel, as well as clipboards and pens with 70% liquid alcohol. Two questionnaires were applied: the first was the Temporomandibular Disorders Symptoms Questionnaire translated into Portuguese (Ohrbach, 2016). This questionnaire consists of 17 questions that assess the presence of jaw pain, history of jaw locks with the mouth open or closed, and the presence of joint noises. In this questionnaire, five points are decisive in the diagnosis of TMD: orofacial pain, headache that extends to the temples, joint sounds, closed mandibular locking and open mandibular locking. The second was the HAD Anxiety and Depression Scale translated into Portuguese (Botega et al., 1995). It consists of 14 questions that assess symptoms of anxiety and depression and it has an evaluation score: Anxiety (Without anxiety from 0 to 8 and With anxiety ≥ 9) and Depression (Without depression from 0 to 8 and with depression ≥ 9).

### Statistical analysis

Descriptive analyses were performed to verify data consistency. For qualitative variables (categorical), absolute and relative frequency was used. For quantitative (numerical), data, position (mean) and dispersion (standard deviation) measures were used. The data were entered into an electronic spreadsheet in the Microsoft Excel® editor and analyzed using the software Statistical Package for the Social Sciences, version 26. In this work, the usual confidence level of 5% (0.05) was used, in order to perform analysis of the statistical tests, and p-value<0.05 was considered significant, thus, the hypothesis of equality (Ho) was disregarded.

## Results

The total sample consisted of 100 individuals (74% of prisoners and 26% of prison employees) divided into two research groups. The results of the assessment of the HAD scale showed that 15.4% of the employees had anxiety symptoms, while the anxiety rate in prisoners was 64.9%. Regarding depression, the percentage of employees with symptoms was only 11.5%, while the number of prisoners with depression reached 48.6%. Table 1 shows the association between anxiety symptoms and TMD only among prison employees. No statistical significance was found between the results, except for the question about headaches extending to the temples, as 100% of the employees who reported anxiety also complained of headaches in the temporal region.

**Table 1:** Analysis of the association between anxiety symptoms from the HAD questionnaire and TMD symptoms from the DC/TMD questionnaire of penitentiary professionals

	Anxiety			
	
	No	Yes		
	
	N(%)	N(%)	P-value	OR
**Have you ever felt pain in your jaw (mouth), temple, ear, or in front of the ear on either side?**			0.606	
No	14(63.6)	2(50.0)		
Yes	8(36.4)	2(50.0)		
**In the past 30 days, have you had any headaches that reached the temple areas of your head?**			0.030	
No	13(59.1)	0(0.0)		b
Yes	9(40.9)	4(100.0)		-
**In the past 30 days, have you heard any sounds or noises in your joint when you moved or used your jaw (mouth)?**			0.090	
No	19(86.4)	2(50.0)		
Yes	3(13.6)	2(50.0)		
**Has your jaw (mouth) ever locked or hesitated, even for a moment, so that you couldn't open it all the way?**			0.562	
No	19(86.4)	3(75.0)		
Yes	3(13.6)	1(25.0)		
**In the past 30 days, when you've opened your mouth wide, has it stuck or hesitated even for a moment so that you couldn't close it from this wide opening position?**			0.664	
No	21(95.5)	4(100.0)		
Yes	1(4.5)	0(0.0)		

Legend: ^1^Fisher's Exact Test, at the 5% level; ^2^OR adjusted: a djusted odds' ratio, at the 5% level.

When analyzing the association between symptoms of anxiety and TMD only in the arrested individuals, no statistical significance was found (Table 2). When performing the same analysis of association between depression and TMD with prison employees, it was only possible to observe statistical significance in relation to closed locking. Those who had symptoms of depression were 21 times more likely to have symptoms of mandibular closed locking (Table 3). When evaluating the association between depression and TMD with prisoners, statistical significance was also observed only regarding closed locking. Prisoners who have symptoms of depression were 3.5 times more likely to have symptoms of mandibular locking (Table 4).

**Table 2:** Analysis of the association between anxiety symptoms from the HAD questionnaire and TMD symptoms from the DC/TMD questionnaire of prisoners in the penitentiary

	Anxiety		
	
	No	Yes	
	
	N(%)	N(%)	P-value
**Have you ever had pain in your jaw (mouth), temple, ear, or in front of your ear on either side?**			0.541
No	10(38.5)	22(45.8)	
Yes	16(61.5)	26(54.2)	
**In the past 30 days, have you had any headaches that reached the temple areas of your head?**			0.154
No	19(73.1)	27(56.3)	
Yes	7(26.9)	21(43.8)	
**In the past 30 days, have you heard any sounds or noises in your joint when you moved or used your jaw (mouth)?**			0.838
No	19(/3.1)	34(70.8)	
Yes	7(26.9)	14(29.2)	
**Has your jaw (mouth) ever locked or hesitated, even for a moment, so that you couldn't open it all the way?**			0.208
No	18(69.2)	26(54.2)	
Yes	8(30.8)	22(45.8)	
**In the past 30 days, when you've opened your mouth wide, has it stuck or hesitated even for a moment so that you couldn't close it from this wide opening position?**			0.459
No	26(100.0)	47(97.9)	
Yes	0(0.0)	1(2.1)	

Legend: ^1^Fisher's Exact Test, at the 5% level.

**Table 3:** Analysis of the association between depression symptoms from the HAD questionnaire and TMD symptoms from the DC/TMD questionnaire of the penitentiary

	Depression			
	
	No	Yes		
	
	N(%)	N(%)	P-value	OR
**Have you ever had pain in your jaw (mouth), temple, ear, or in front of your ear on either side?**			0.286	
No	15(65.2)	1(33.3)		
Yes	8(34.8)	2(66.7)		
**In the past 30 days, have you had any headaches that reached the temple areas of your head?**			0.066	
No	13(56.5)	0(0.0)		
Yes	10(43.5)	3(100.0)		
**In the past 30 days, have you heard any sounds or noises in your joint when you moved or used your jaw (mouth)?**			0.369	
No	18(78.3)	3(100.0)		
Yes	5(21.7)	0(0.0)		
**Has your jaw (mouth) ever locked or hesitated, even for a moment, so that you couldn't open it all the way?**			**0.009**	
No	21(91.3)	1(33.3)		b
Yes	2(8.7)	2(66.7)		21,000(1,271-346.934)
**In the last 30 days, when you opened your mouth wide, did it lag or hesitate even for a moment so that you were unable to close it from this wide opening position?**			0.713	
No	22(95.7)	3(100.0)		
Yes	1(4.3)	0(0.0)		

Legend: ^1^Tisher's Exact Test, at the 5% level; ^2^OR adjusted: a djusted odds' ratio, at the 5% level.

**Table 4:** Analysis of the association between symptoms of depression in the HAD questionnaire and symptoms of TMD in the DC/TMD questionnaire of prisoners

	Depression			
	
	No	Yes		
	
	N(%)	N(%)	P-value	OR
**Have you ever had pain in your jaw (mouth), temple, ear or in front of your ear on either side?**			0.790	
No	17(44.7)	15(41.7)		
Yes	21(55.3)	21(58.3)		
**In the past 30 days, have you had any headaches that reached the temple areas of your head?**			0.254	
No	26(68.4)	20(55.6)		
Yes	12(31.6)	16(44.4)		
**In the past 30 days, have you heard any sounds or noises in your joint when you moved or used your jaw (mouth)?**			0.686	
No	28(73.7)	25(69.4)		
Yes	10(26.3)	11(30.6)		
**Has your jaw (mouth) ever locked or hesitated, even for a moment, so that you couldn't open it all the way?**			**0.010**	
No	28(73.7)	16(44.4)		b
Yes	10(26.3)	20(55.6)		3,500(1,318-9,293)
**In the past 30 days, when you've opened your mouth wide, has it stuck or hesitated even for a moment so that you couldn't close it from this wide opening position?**			0.301	
No	38(100.0)	35(97.2)		
Yes	0(0.0)	1(2.8)		

Legend: ^1^Fisher's Exact Test, at the 5% level; ^2^OR adjusted: adjusted odds ratio, at the 5% level.

## Discussion

In the present study, TMD symptoms were evaluated in both prisoners and employees, and the results could be compared. The TMD symptoms most found in prison workers were: headache extending to the temporal region (50%), orofacial pain (38.5%), and joint noises (19.2%). These findings are similar to others TMD symptoms related in prisoners in other studies (Enguelberg-Gabbay et al., 2016; Vainionpää et al., 2018). Several studies have evaluated the prevalence of depression and anxiety in prisoners from different countries around the world: Turkey (Unver et al., 2013), Italy (Cavallo et al., 2014; D'aurizio et al., 2020), Nigeria (Osasona, Koleoso, 2015), England (López-Pérez et al., 2018; Packham et al., 2020; Butcher et al., 2021), Scotland (Arora et al., 2020), Brazil (Ranuzi et al., 2020), Poland (Stawinska-Witoszynska et al., 2021), and Ethiopia (Abdulkadir et al., 2022). None of these researchers investigated symptoms of anxiety and depression in professionals working in the same penitentiaries, c ontrary to this present study, in which it was observed that 15.4 % of employees had symptoms of anxiety and 11.5 % had symptoms of depression. Different types of questionnaires were used to investigate the presence of anxiety and depression with different parameters: DASS-42 (Unver et al., 2013), EQ-5D (Cavallo et al., 2014), HAD (Osasona, Koleoso, 2015), CES-D (López-Pérez et al., 2018; Arora et al., 2020), GAD-7 (López-Pérez et al., 2018; Packham et al.; 2020; Butcher et al., 2021), PHQ -9 (Packham et al., 2020; Butcher et al., 2021; Abdulkadir et al., 2022), BDI-2 (D'aurizio et al., 2020), STAI (D'aurizio et al., 2020), and DASS-21 (Ranuzi et al., 2020). One of the studies investigated more comprehensively the anxiety and depression symptoms of prisoners through their prison medical records, pre-incarceration medical records, the results of medical consultations outside prison, and the results of complementary tests (Stawinska-Witoszynska et al., 2021). The HAD scale was chosen to be used in the present study because it is a short questionnaire, which can be filled in just a few minutes, used in non-psychiatric environments, it is easy to understand, and the answers are based on how the individual felt during the previous week. It is possible to notice that the mentioned literature is limited. The reason behind this is that usually research in prisons are rare due to bureaucracy, but also to the great possibility of encountering various obstacles during the process, such as violence (Unver et al., 2013; Cavallo et al., 2014; Osasona, Koleoso, 2015; Enguelberg-Gabbay et al., 2016; López-Pérez et al., 2018; Vainionpää et al., 2018; Arora et al., 2020; D'aurizio et al., 2020; Packham et al., 2020; Butcher et al., 2021; Stawinska-Witoszynska et al., 2021; Abdulkadir et al., 2022).

The number of prisoners with anxiety, according to the HAD scale, was considered high in this study (64.9%). Other studies showed different results: 61.9% (Unver et al., 2013), 77.8% (Osasona, Koleoso 2015), 59.1% (López-Pérez et al., 2018), 31.5% (Packham et al., 2020), 49.4% (Butcher et al., 2021) and 6.9% (Stawinska-Witoszynska et al., 2021). Regarding depression, according to the HAD scale, in this study it was found that 48.6% of the prisoners had depressive symptoms. The referenced literature indicates a variation between 1.7 and 72.6% (Unver et al., 2013; Osasona, Koleoso 2015; López-Pérez et al., 2018; Arora et al., 2020; Packham et al., 2020; Ranuzi et al., 2020; Butcher et al., 2021; Stawinska-Witoszynska et al., 2021; Abdulkadir et al., 2022). Anxiety and depression levels were also assessed in prison workers with results of 15.38% and 11.53%, respectively. Counterweighting the levels found in prisoners of the same prison, 64.86% for anxiety and 48.64% for depression, the levels of the workers are significantly lower. This shows that the prison environment affects the prisoners' mental health much more than the employees' mental health. The present study observed important values in the number of prisoners with symptoms of orofacial pain (56.75%), headaches that includes the temporal region (37.83%), joint noises (28.37%), and jaw locking (40.54%). Only in the open locking of the mandible item, the rate was low (1.35%). In Finland, a study using the same questionnaire had similar results in terms of orofacial pain (54%) and headache that includes the temporal region (37%), with much higher results in terms of joint noise (43%) and open locking of the mandible (8%), and a much lower value when referring to closed mandibular locking, with only 7% (Vainionpää et al., 2018). Another study showed that 46.3% of drug-using prisoners had TMD symptoms, while the result among non-drug users was only 25.6% (Enguelberg-Gabbay et al., 2016). Although the results are close to those found in the present study, it did not distinguish between drug users and non-users. Further conclusions come up against the limitations of the present study, which is a preliminary analysis of a sample. Access to medical records of prisoners prior to imprisonment, if they exist, could show us whether symptoms of anxiety, depression, and TMD were already present. There are few studies that have evaluated symptoms of anxiety and depression in prisoners in Brazil, and the results can be worrying, which indicates the need for further studies in prisons in different locations.

## Conclusion

It can be concluded that prison life affects prisoners and staff differently with regard to anxiety, depression, and TMD. It is clear that prisoners suffer much more from anxiety and depression than employees of the same prison.
